# The Economic Impact of Early Life Environmental Tobacco Smoke Exposure: Early Intervention for Developmental Delay

**DOI:** 10.1289/ehp.9165

**Published:** 2006-07-11

**Authors:** Thaddeus Miller, Virginia A. Rauh, Sherry A.M. Glied, Dale Hattis, Andrew Rundle, Howard Andrews, Frederica Perera

**Affiliations:** 1 Columbia Center for Children’s Environmental Health, Mailman School of Public Health, Columbia University, New York, New York, USA; 2 Health Policy & Management, Columbia University, New York, New York, USA; 3 George Perkins Marsh Institute, Clark University, Worcester, Massachusetts, USA

**Keywords:** asthma, children, developmental delay, environmental tobacco smoke

## Abstract

**Background and Objectives:**

Early-life exposure to environmental tobacco smoke (ETS) can result in developmental delay as well as childhood asthma and increased risk of cancer. The high cost of childhood asthma related to ETS exposure has been widely recognized; however, the economic impact of ETS-related developmental delay has been less well understood.

**Methods and Results:**

The Columbia Center for Children’s Environmental Health (CCCEH) has reported adverse effects of prenatal ETS exposure on child development in a cohort of minority women and children in New York City (odds ratio of developmental delay = 2.36; 95% confidence interval 1.22–4.58). Using the environmentally attributable fraction (EAF) approach, we estimated the annual cost of one aspect of ETS-related developmental delay: Early Intervention Services. The estimated cost of these services per year due to ETS exposure is > $50 million per year for New York City Medicaid births and $99 million per year for all New York City births.

**Conclusion:**

The high annual cost of just one aspect of developmental delay due to prenatal exposure to ETS provides further impetus for increased prevention efforts such as educational programs to promote smoke-free homes, additional cigarette taxes, and subsidizing of smoking cessation programs.

An estimated 35–80% of inner-city children are exposed to environmental tobacco smoke (ETS) (DiFranza et al. 2004; Gergen et al. 1998; Hopper and Craig 2000; Kum-Nji et al. 2006; Weaver et al. 1996). The health effects associated with early-life ETS exposure include reduced fetal growth, neurodevelopmental problems, respiratory disease including asthma, increased cancer risk, and cardiovascular disease, with some adverse effects manifesting in childhood and others in adulthood (Samet 2005; U.S. Department of Health and Human Services 2006).

In earlier work, we documented an association between ETS and developmental delay (Rauh et al. 2004). Here we present estimates of the cost of Early Intervention Services to remediate these developmental delays attributable to ETS in the New York City Medicaid population and in the total New York City population. In the first step, based on the CCCEH results (Rauh et al. 2004), we calculated the fraction of total delay attributable to mothers’ ETS exposure during pregnancy (the environmentally attributable fraction, or EAF). The EAF was then multiplied by the prevalence of delay, the size of the exposed population, and the cost per intervention to give the estimated annual cost of Early Intervention Services among children with ETS-related developmental delay. We find that there are substantial costs for the interventions needed to address ETS-related developmental delay. These costs provide more impetus to efforts on ETS prevention. Finally, we describe feasible approaches to intervention.

## Health Effects of ETS Exposure

With respect to developmental effects, there is no evidence of a threshold below which ETS exposure is safe (DiFranza et al. 2004; Samet 2005). For example, Martin and Bracken (1986) reported a doubling in the risk for low-birth-weight babies when the nonsmoking mother was exposed to ETS for ≥ 2 hr/day. Based on meta-analysis, Windham et al. (1999) estimated that passive maternal smoking during pregnancy results in an average decrease of 31 g in birth weight. The Ottawa Prenatal Prospective Study reported that the long-term effects of maternal passive smoking during pregnancy were smaller in magnitude but qualitatively similar to those of active maternal smoking and included effects on speech and language skills, intelligence, visual/spatial abilities and mother’s rating of behavior (Makin et al. 1991). Other studies have also found an inverse relationship between prenatal ETS exposure and indicators of developmental delay, such as on the Bayley Mental Development Index (MDI) and the Wechsler Intelligence Scale for Children (Rauh et al. 2004; Yolton et al. 2005).

With respect to nondevelopmental effects, ETS exposure in childhood has long been established as a “trigger” for asthma in children (Gergen et al. 1998; Mannino et al. 2002). Nationally, a 1992 report by the U.S. Environmental Protection Agency (EPA) attributed 200,000 of 1,000,000 cases of childhood asthma exacerbation to secondhand smoke (U.S. EPA 1992). Based on nationally representative data from 1988–1994, Gergen et al. (1998) estimated that between 40 and 60% of the cases of asthma among children 2 months to 5 years of age who were exposed to ETS are attributable to ETS exposure. Some studies have found an association between early-life ETS exposure and childhood and adult cancer, although results have been inconsistent (Bofetta 2000; Janerich et al. 1990; Ji et al. 1997; Sasco and Vainio 1999; Sorahan et al. 1997; Stockwell et al. 1992; Vineis et al. 2005).

In addition to the main effects of ETS on early development, there is some evidence of synergistic interactions between ETS and air pollutants and between ETS and psychosocial stress, such that the co-occurrence of ETS with either of the two other exposures multiplies the risk of adverse effects. In the CCCEH prospective cohort in New York City, such an interaction effect of ETS and polycyclic aromatic hydrocarbons (PAH)–DNA adducts was observed on birth weight and head circumference (Perera et al. 2004). In addition, the impact of ETS on 2-year cognitive development was exacerbated by material hardship (Rauh et al. 2004). In the same cohort study, evidence showed an interaction between exposure to prenatal PAHs and postnatal ETS on increased risk of respiratory problems in children (Miller et al. 2004). Such observed interactions suggest that health-related costs associated with exposure to secondhand smoke may be increased when other “toxic” exposures co-occur.

The fetus and young child are particularly susceptible to the effects of ETS and its chemical constituents, including PAHs (Perera et al. 2004). For example, several studies of paired cord and maternal blood taken at delivery have shown higher levels of PAH–DNA adducts per estimated unit of exposure (Perera et al. 2004; Whyatt et al. 2001). Significantly higher cotinine levels have also been observed in cord blood compared to maternal samples, probably as a consequence of a slower rate of clearance of this nicotine metabolite from the fetal system (Perera et al. 2004; Whyatt et al. 2001).

## ETS-Related Developmental Delay and Some Estimated Costs

### The CCCEH Cohort Study: ETS-related developmental delay

The cohort study includes African-American and Latina mothers enrolled during pregnancy and their children. Women who reported active smoking during pregnancy were excluded, as were women who reported diabetes, hypertension, positive HIV status, or drug or alcohol abuse, or whose previous babies were low birth weight.

The analysis of the risk of ETS for adverse child development involved 226 infants from the CCCEH cohort who had reached 24 months of age at the time of the analysis and had complete data on all measures (Rauh et al. 2004). ETS exposure was self-reported and cotinine-verified. Prenatal ETS exposure occurred in 40.2% of the children. Child development was measured using the Mental Development Index (MDI) of the Bayley Scales of Infant Intelligence. The MDI is a useful measure of mental/cognitive performance when administered after 18 months of age (Slater 1995). Several studies confirm the ability of the MDI to identify children who will demonstrate developmental delay on tests of school-age ability and achievement measures (e.g., Siegel 1979; Singer and Fagan 1984).

Because estimation of the effects of ETS exposure on neurodevelopment can be complicated by confounding, inaccurate assessment of exposure, colinearity of postnatal ETS exposure, and other prenatal maternal exposures (Eskenazi and Castorina 1999), the regression models adjusted for the effects of race/ethnicity, sex, gestational age (weeks), age at test administration, maternal education, marital status, material hardship, and postnatal ETS exposure. Self-reported prenatal ETS exposure was validated with a biomarker (cotinine levels in cord blood). The models tested the effects of ETS among women with and without material hardships. There was a significant negative impact of ETS (*p* = 0.005), resulting in an estimated mean (± SE) 4.8 ± 1.6 point average deficit in MDI at 24 months of age and an odds ratio of 2.36 for developmental delay [95% confidence interval (CI), 1.22–4.58]. A separate model indicated a significant interaction between ETS and material hardship (*p* = 0.03), such that children who were exposed to both ETS and material hardship had an even greater average cognitive deficit of 7.1 ± 3.3 points (Rauh et al. 2004). It is likely that the effects of ETS on development were underestimated because women who actively smoked or used illicit drugs were ineligible for enrollment, and the study design eliminated most preterm deliveries (Rauh et al. 2004).

With a Bayley MDI score < 80 to categorize developmental delay, the CCCEH cohort has a high rate of developmental delay (32.3% of the children in the cohort). However, this rate is comparable to that found in other predominantly low-income New York samples. For example, in a sample of 12- to 36-month-old low-income children attending a pediatric ambulatory clinic in New York, Dreyer et al. (1996) reported that 49% had Bayley MDI scores of ≤ 85, and 9% had scores < 70. In a recent study of Hispanic and African-American 2-year-olds in the South Bronx, 30% had Bayley MDI scores < 70 (Huberman et al. 2003), whereas 17.9% of the children in the CCCEH cohort scored < 70 on the Bayley MDI.

Unlike most prior studies, the CCCEH cohort study clearly differentiated the effects of prenatal and postnatal ETS exposure. The results were consistent with a prior study of the cognitive effects of ETS exposure that reported that children of mothers exposed to ETS during pregnancy scored below the unexposed group on the Peabody Picture Vocabulary Test, Perceptual Organization, and Freedom from Distractibility, and on measures of language, intelligence, and attention (Makin et al. 1991).

### Calculation of cost-related Early Intervention Services

To calculate the costs of developmental deficits attributable to prenatal ETS exposure, we employed the general model (Trasande et al. 2005):


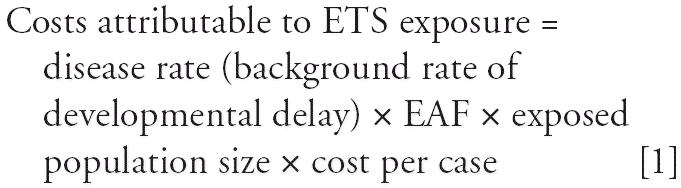


The disease rate (25.9%) was based on data from the CCCEH study described above (Rauh et al. 2004). The EAF method was developed by the Institute of Medicine (Institute of Medicine 1981), and has been widely used to calculate the environmentally attributable costs of pediatric cancer, asthma, lead poisoning, neurodevelopmental disabilities, and methylmercury poisoning (Landrigan et al. 2002).

The EAF is a composite value and is the product of the prevalence of ETS exposure (fraction of population exposed) and the relative risk of delay associated with exposure. In the CCCEH study, the observed odds ratio for developmental delay was 2.36 (95% CI, 1.22–4.58). Because the odds ratio overestimates the estimated relative risk when the outcome is common (Rothman 1996), based on data from Rauh et al. (2004), the relative risk of 1.61 (95% CI, 1.11–2.34) was used to calculate the EAF. The “exposed population size” is the population at risk (i.e., exposed to ETS) and was estimated in two ways: *a*) based on the number of New York City children with characteristics similar to those in the CCCEH cohort (i.e., Medicaid births in New York City in 2002); and *b*) based on the total number of New York City births in 2002 (New York City Department of Health and Mental Hygiene 2002). Our first analysis used Medicaid births in New York City as a comparable population because > 90% of the births in the CCCEH were to mothers on Medicaid. The prevalence of exposure in both populations was estimated to be 40.2% based on the CCCEH data and other studies (Gergen et al. 1998; Pirkle et al. 1996; Rauh et al. 2004). A prevalence of exposure of about 40% is likely to be an underestimate for the Medicaid births because the CCCEH cohort, by design, includes nonsmokers, and inner-city exposure rates have been shown to be as high as 70–80% (DiFranza et al. 2004; Gergen et al. 1998; Pirkle et al. 1996; Weaver et al. 1996). Our second analysis extrapolated to all New York City births to calculate an upper estimate for the costs of Early Intervention Services. The rationale was that, according to the literature, the adverse developmental effects of prenatal ETS exposure are seen in diverse populations and are not limited to Medicaid births (Makin et al. 1991; Rauh et al. 2004; Yolton et al. 2005).

The “cost per case” is the average cost per child for Early Intervention Services over the average time of enrollment (provided by the New York City Department of Health and Mental Hygiene, Early Intervention Program, personal communication). U.S. law mandates that Early Intervention Services be provided to those children at risk of developmental delay (Kirby et al. 1993). The Early Intervention Program consists of home-based services provided to the family who elects to participate, with a typical child receiving about four contacts per week. Developmentally delayed children are defined as having an MDI < 80 at 24 months of age and are referred to the Early Intervention Program for screening and services. Among all New York City children who were referred to Early Intervention, an average of 70% were evaluated, found to be eligible, and participated in the service program between July 2003 and June 2004 (New York City Department of Health and Mental Hygiene, Early Intervention Program, personal communication). Citywide, the total cost for Early Intervention Services amounted to slightly over $443 million for 39,000 children participating during the 1-year period between July 2003 and June 2004 for annual cost per case of approximately $11,500 (New York City Department of Health and Mental Hygiene, Early Intervention Program, personal communication). Each child is enrolled for an average of 18 months, which costs approximately $17,200. We take this as our “cost per case” (Table 1 summarizes the data used in the health cost calculation).

Table 2 summarizes the estimated annual costs of ETS-related Early Intervention for New York City Medicaid births and for all New York City births in 2002. These costs ($51.5 million and $99 million, respectively), would accrue each year as “new” children who were exposed *in utero* to ETS become eligible for enrollment. So, for example, over a 10-year period the present value of the costs for Early Intervention Services attributable to prenatal ETS exposure among New York City Medicaid births would be approximately $439 million (based on a 3% annual discount rate). Although the estimated costs of Early Intervention are significant, they represent only a fraction of all the costs of developmental delay due to ETS exposure. Early Intervention programs do not capture all those children who are delayed, leaving a substantial proportion of cases undetected and unaddressed by health providers and early childhood programs (Shonkoff and Mesiels 2000). Nor do Early Intervention programs ameliorate all the effects of developmental delay for those enrolled.

Costs from residual developmental problems not corrected by Early Intervention could include those of some Special Education services. In 1999–2000, the average cost for each student in special education was $12,500 versus $6,500 for a non-special-education student (National Association of State Boards of Educations 2002). In addition, grade retention costs about $6,000 per year per student (Shepard and Smith 1990). In a cost–benefit analysis of the Perry Preschool Project, for every dollar invested in early intervention, savings of approximately $7 are accrued as a result of decreased rates of special education and grade retention among the intervention group (Barnett 1985). Grade retention, in turn, is predictive of the likelihood of graduating from high school, which serves as an important predictor of socioeconomic well-being and health status (Anderson et al. 2003). Thus, the prevention of ETS exposure can be viewed as an investment good that yields personal and social benefits into the future.

## Discussion

This analysis highlights the substantial burden of developmental impairment and illness associated with prenatal and postnatal ETS exposure. We estimate that the annual cost of Early Intervention Services for developmental delay attributable to ETS exposure in New York City is slightly over $51 million for New York City Medicaid births and $99 million for all New York City births.

The costs estimated here represent just one element of the costs of developmental delay associated with ETS. The economic costs of postnatal ETS also include costs of developmental delay that cannot be remediated through early intervention

This analysis adds to the other evidence of substantial costs associated with other health effects of ETS. In particular, postnatal ETS exposure is a well-known trigger for childhood asthma (Gergen et al. 1998; Mannino et al. 2002); and the high cost of childhood asthma has been widely recognized (American Legacy Foundation 2004; Sullivan et al. 2005; Wang et al. 2005) For example, among children 2 months to 5 years of age, an estimated 133,800–161,600 excess cases of asthma in the U.S. are attributed to ETS exposure (Gergen et al. 1998). The estimated cost of pediatric asthma care per case ranges from $385/year for usual care (Sullivan et al. 2005) to $791 including parent’s loss of productivity due to children’s asthma-related school absence days (Wang et al. 2005). Some evidence also suggests a causal role of ETS in cancer in childhood or adulthood (Bofetta 2000; Janerich et al. 1990; Sorahan et al. 1997; Stockwell et al. 1992; Vineis et al. 2005). The established link between postnatal ETS and asthma and the potential link between ETS and cancer suggest that in the New York City population there are likely to be substantial economic costs associated with these consequences of ETS as well. Thus, the costs described above likely represent only a fraction of the true costs of ETS-related effects.

The limitations of this analysis should be noted. First, because the CCCEH cohort is comprised of nonsmoking, healthy women, the relative risk and EAF based on cohort data may underestimate the risk to the Medicaid population. Second, in extrapolating the costs of Early Intervention to the New York City population we were unable to conduct a finer analysis, because we did not have data on the effects on development according to median household income or other variables such as maternal education. Third, these analyses are based on observational data and thus are subject to confounding by unobservable risk factors.

The data support stronger efforts to prevent ETS exposure to pregnant women and children. Many cities and states have passed smoke-free legislation, including New York City; Albuquerque, New Mexico; Maine; and Connecticut. Federal, state, and local legislation has been effective in reducing secondhand smoke exposure in the workplace and public places. In fact, nonsmokers’ exposure to tobacco smoke has fallen by > 70% from 1988–1991 to 1999–2000 (National Cancer Institute 2005; Pirkle et al. 2006). However, there is still a very high prevalence of ETS exposure among U.S. children, ranging from 35 to 80% depending on the method of measurement used and the population studied (Kum-Nji et al. 2006); and risks from smoking in the home remain.

Several approaches have been effective in reducing ETS exposure in the home (Emmons et al. 2001; Hovell et al. 2000; Okah et al. 2002). The first approach is counseling by health professionals and the promotion of home smoking restrictions: a “rule of the house” that forbids smoking inside the home. Only 12.5–25% of homes with smokers restrict smoking; and families of lower socioeconomic status are even less likely to have home restrictions (Norman et al. 1999). Reason for this low rate could be insufficient information on the risks of ETS to infants and children and the lack of professional counseling. For example, when mothers were counseled by health professionals about the dangers of passive smoke exposure, there was a dramatic reduction in children’s exposure to cigarettes per week—by almost 90% over 12 months (Hovell et al. 2000). Similarly, a study targeting low-income families with young children found that parents reduced their child’s ETS exposure after training and follow-up counseling (Emmons et al. 2001). However, barriers to the adoption of home smoking restriction, such as male dominance in the family, must be addressed.

A second approach to changing smoking behavior and reducing children’s exposure to ETS is through market interventions, including an increased tax on cigarettes to cover the external health costs generated by ETS exposure.

Another potential intervention is government subsidizing of smoking cessation programs. Such programs could be targeted to smokers in households with or expecting children. Smoking cessation programs range from brief advice and counseling, to nicotine replacement therapy (e.g., nicotine patches), to intensive clinical interventions including bupropion therapy (Parrott and Godfrey 2004). A major appeal of government subsidizing of smoking cessation is the cost-effectiveness of cessation programs (Parrott and Godfrey 2004). The Agency for Health Care Policy and Research (2005) estimated that the average cost per smoker undergoing a smoking cessation intervention is $165.61. This can be compared to the substantial health costs to the smoker as well as the smokers’ families. If such programs were targeted to New York City families, the total costs of smoking cessation would be only 8.7% of the potential costs of Early Intervention Services for children developmentally delayed due to their mothers’ passive maternal smoke exposure during pregnancy.

## Conclusion

The present analysis shows that the economic costs of developmental delay due to prenatal ETS exposure are substantial. Adding to a large body of evidence on the health, social, and economic costs of ETS, these data provide additional impetus for interventions to protect this vulnerable population.

## Figures and Tables

**Table 1:** Data for the health cost calculation.

	Value
Delay rate (%)^a^	25.9
Prevalence of exposure (%)^a^	40.2
EAF^a^	0.647
Exposed population size, New York City (2002) (*n*)	
Medicaid births, exposed^b^	25,500
All births, exposed	49,400
Cost per case^c^	$17,200

a Rauh et al. 2004.

b Number of births from New York City Department of Health and Mental Hygiene (2002).

c New York City Department of Health and Mental Hygiene, Early Intervention Program (personal communication).

**Table 2:** Estimated annual costs of ETS-related developmental delay, 2004: Early Intervention Services for New York City Medicaid births and all New York City births.

	New York City Medicaid births^a^	All New York City births^a^
No. of live births	63,462	122,937
No. exposed	25,500	49,400
No. delayed due to ETS	4,300	8,300
Remedial intervention	Early Intervention	Early Intervention
No. enrolled in Early Intervention	3,000	5,800
Total cost	$51.5 million	$99 million

a Based on data from New York City Department of Health and Mental Hygiene (2002).
